# Exploring the views of people experiencing homelessness regarding their health and housing support, and their perspectives on deteriorating health and end of life support: a qualitative analysis

**DOI:** 10.1136/bmjopen-2026-121183

**Published:** 2026-10-09

**Authors:** Samantha Dorney-Smith, Joel Barnett, Sarah Hough, Joanne Kennedy, Sahar Khan, Pete Bull, Gareth Davis, Mandy Pattinson, Caroline Shulman, Briony Hudson

**Affiliations:** 1 UCL, London, UK; 2 Pathway, London, UK; 3 Groundswell, London, UK; 4 Marie Curie, London, UK

**Keywords:** Health Equity, Substance misuse, MENTAL HEALTH, PALLIATIVE CARE, MEDICAL EDUCATION & TRAINING

## Abstract

**Abstract:**

**Objectives:**

To explore the views of people with lived experience of homelessness regarding health, housing and social care support and the support people with deteriorating health may need.

**Design, settings and participants:**

14 qualitative interviews were undertaken with people experiencing homelessness who were in contact with services that had previously participated in a Community of Practice intervention. Participants resided in hostels (n=12) or supported housing (n=2). Interviews were conducted primarily face to face and were supported by a lived experience researcher. Data were analysed using reflexive thematic analysis and were used to inform the refinement of the Community of Practice intervention.

**Results:**

Four themes were identified: the impact of homelessness on health; structural and relational barriers to equitable healthcare; contribution of the hostel environment to poor health; views on how health and homelessness service staff could respond to deteriorating health.

A high burden of health issues, psychological trauma and exposure to death were described. Health and social care services were perceived as absent or hard to access. Mental health support and adequate pain control were challenges. Stigmatising experiences in hospital, often related to addiction, were common.

Interviewees supported more proactive conversations around deteriorating health.

**Conclusion:**

The risks and reality of deteriorating health among people experiencing homelessness were widely recognised. Interviewees felt that sensitive conversations about this issue could be initiated by staff. However, healthier environments and improved access to health and care support were needed.

Lived experience input was a vital component for the development of the Community of Practice programme.

**STRENGTHS AND LIMITATIONS OF THIS STUDY**

- This work and the wider Intervention to Optimise Palliative Care for People with Lived Experience of Homeless (IMPROVE) study are supported by a 5-person lived experience steering group.
- The majority of interviews were conducted with a lived experience interviewer.
- In terms of wider relevance, the number of interviewees (n=14) and participating hostels (n=3) was small. There were also more men (n=11) than women (n=3) interviewed, although this does reflect the common gender balance seen in many homelessness services. By chance, all interviewees were White British.
- The participating hostels were also larger than average (between 50–90 beds), so experiences in smaller hostels may differ.

## Introduction

 People experiencing homelessness are known to have high health needs^1^ and significantly increased mortality ratios.^2^ They are also known to have challenges accessing health and social care services^3–6^ and appropriate housing and support.^4 6^ These barriers in accessing healthcare often persist towards the end of life. Barriers to the identification of palliative care needs and accessing support include a lack of a formal diagnosis and the early age at which conditions are experienced.^7–9^ This is often because many of the life-limiting conditions involved, for example, liver disease, can be related to addictions and may therefore be potentially reversible with behaviour change. This can make the prediction of outcomes and illness trajectories difficult.^7–9^

In the UK, palliative care is delivered through a mixed model involving generalist health and social care providers, specialist palliative care teams, hospices, community services, hospitals, care homes and voluntary sector organisations. Inequity in palliative care access is well evidenced in the literature^10 11^ due to the high level of complexity and unmet social needs; mainstream palliative care services such as hospices, community nursing services and general practitioners can be unsure how to support people who are experiencing homelessness.^7 8^ In addition, the homelessness services directly supporting clients often have limited awareness of the potential benefits of a ‘palliative approach’ and advance care planning.^8^ Similar challenges have been observed in studies conducted in the Netherlands,^12^ Canada^13 14^ and America.^15^

These interviews aimed to explore the views of people living in temporary accommodation in regard to the health and housing support needs of people experiencing homelessness whose health may be declining and who may therefore be approaching the ends of their lives.

This work was undertaken to further inform the content of the Intervention to Optimise Palliative Care for People with Lived Experience of Homeless^16^ (IMPROVE), which is a Community of Practice intervention focused on improving care for people experiencing homelessness with palliative care needs. The intervention focuses on how to optimise support for people experiencing homelessness with advanced ill health, who may benefit from a palliative approach, by connecting professionals from different backgrounds who work in the same town or city. These professionals attend a series of eight online sessions which promote the exchange of knowledge and best practice around various aspects of palliative care for people experiencing homelessness.

A discreet set of 14 qualitative lived experience interviews were undertaken, as part of a larger project focused on developing and refining the content for the IMPROVE.^16^ 33 practitioner interviews were also undertaken. IMPROVE is the second iteration of a Community of Practice intervention described above, that was piloted in four sites in three areas across the UK between 2020 and 2021.^17^ The current project to optimise this intervention began, IMPROVE, began in November 2024.

Within IMPROVE ‘a palliative approach’ is promoted.^18^ This includes a focus on ‘parallel planning’^19^ and improving the quality of life for individuals by managing their symptoms, providing emotional, social and spiritual support, and meeting their practical needs. This is presented as something which can be delivered by a variety of partners, and not just specialist palliative care services. This article outlines the themes identified from the interviews, and the implications for creating content for the IMPROVE programme, as well as the wider messages for people from a range of professional backgrounds who support people experiencing homelessness.

### Aims

The interviews aimed to explore the following questions:

1. What do people with a lived experience of homelessness think health, housing, social care or voluntary sector professionals need to understand when supporting people who are currently homeless and who have deteriorating health problems?
2. What do people with a lived experience of homelessness think services could do differently to better meet the needs of people experiencing homelessness with deteriorating health problems?
3. How do people experiencing homelessness feel conversations with people with deteriorating health conditions who might be headed towards the end of life can be approached?

### Methodology

The 14 interviews reported in this paper were undertaken alongside 33 practitioner interviews (reported elsewhere) with the primary purpose of informing the content of the IMPROVE programme.

### Intervention to Optimise Palliative Care for People with Lived Experience of Homelessness (IMPROVE)

The Community of Practice intervention on which IMPROVE is based was initially developed by BH and CS in 2020 and piloted across four sites between 2020 and 2021.^17^ Following this pilot phase, the intervention was implemented across an additional 11 sites between 2023 and 2024. In 2024, funding from the National Institute for Health and Care Research (NIHR) supported further refinement of the intervention, to create the IMPROVE intervention.^20^ Data in this paper are drawn from interviews with the users of homelessness services that participated in original pilot sites and the 11 subsequent implementation sites.

The NIHR funded project to develop IMPROVE is supported by a lived experience group who advise on all aspects of the study. The group felt that the findings of these 14 interviews were so strongly relevant beyond IMPROVE, for example, to people in commissioning and homelessness services, that they warranted their own article. As such, this article has been written with both academic and operational audiences in mind.

Data for the interviews are reported in line with Reflexive Thematic Analysis Reporting Guidelines (RTARG)^21^ and the Consolidated Criteria for Reporting Qualitative Research (COREQ).^22^

### Researcher positioning

An interpretivist approach guided the analysis of the interviews, recognising that individuals interpret and make meaning of their experiences in diverse ways.^23^ Our analysis aimed to understand how interviewees constructed meaning around the questions related to the health and support for people with declining health in the context of homelessness, rather than to identify a single underlying reality.

SD-S is a female Clinical Research Associate and homeless and inclusion health nurse with 24 years’ experience of working directly with people experiencing homelessness and other excluded groups. Inclusion health nurses work to support socially disadvantaged individuals to access the healthcare they need. SH is a female Research Officer for Groundswell, an organisation which aims to raise the voice of people with a lived experience of homelessness through research and activism. SH has lived experience of homelessness. While both researchers are female, SD-S and SH have considerable experience of interviewing people experiencing homelessness across the gender spectrum.

JB is a male Master’s Psychology graduate and Research Fellow, with experience of working in a drug and alcohol recovery charity. He shared the core data analysis and write-up with SD-S. BH and CS are female senior researchers in the specific area of homelessness and palliative care. They conceptualised the research and provided senior oversight throughout.

JK, SK, PB and GD are members of the lived experience group for this project. Their input is described later. MP is the Lived Experience Lead for Pathway, the homeless health charity which is supporting the IMPROVE lived experience group.

### Research participants and settings

#### Inclusion criteria

For the purposes of this article a ‘person with a lived experience of homelessness’ refers to a person who has direct, personal and first-hand experience of not having a stable, safe or permanent home. This includes, for example, rough sleeping, hostel dwelling, temporary housing or sofa-surfing.

Participants were primarily recruited via homelessness services that had participated in the pilot phase of the Community of Practice intervention. To be eligible for inclusion in this study, they also had to have self-reported ‘high health needs,’ be over 18 and be able to provide informed consent to participate. Participants were not required to have a palliative diagnosis to take part in this research.

#### Setting

Interviewees lived in five distinct locations across the UK. The majority (n=12, 86%) lived in one of 3 homelessness hostels. All three hostels were run by voluntary sector organisations on contract to their Local Authority (the part of the UK government that runs services in a specific geographical area of the country, eg, a county or borough) (table 1).

**Table 1:** Homelessness hostels

	No of beds	Gender
Hostel 1	55 beds	Male only
Hostel 2	50 beds	Mixed
Hostel 3	89 beds	Male only

The two other interviewees were living alone, but with support—one in a ‘Housing First’ placement^24^ and one in supported housing—both after a prior history of hostel dwelling and rough sleeping. Housing First placements provide permanent, self-contained housing with no preconditions, alongside flexible support to people with a long history of homelessness. In contrast, homeless hostels offer temporary single bedroom, shared facility supported accommodation, where access and continued stay are typically contingent on meeting basic behavioural or engagement requirements.

All the placements mentioned above require eligibility for welfare support in the UK.

#### Recruitment

The majority of the interviewees were users of homelessness services that participated in the pilot of the Community of Practice intervention (n=12, 86%) and were recruited with support from hostel workers, from within the three hostels.

The researchers worked with hostel staff to identify a date that would be suitable for them to attend the service and conduct the interviews. Hostel workers shared information about the study with service users via posters which were displayed in the hostels. They were also asked to highlight the project to service users with high health needs that they thought might be interested during key working sessions within the 2 weeks prior to the interview day. Hostel workers provided verbal information and a Participant Information Sheet in advance to service users that indicated an interest in participating. Hostel staff compiled a list of people who expressed an interest and contacted them on the day of the interviews to see if they would like to meet with the researchers.

Two participants (n=2, 14%) were previously known to SD-S through their involvement with prior research studies and were purposefully sampled for their insights on this topic. On the days SD-S and SH went to the hostels, all but two of the people who had said they wanted to be interviewed were available, and everyone that was available on the day was interviewed. It is not known how many other service users who were approached declined to participate or what their reasons for not participating were.

#### Data collection

The qualitative semi-structured interviews were undertaken between March and May 2025. Semi-structured interviews were undertaken to allow themes to develop from within the interviews.

Most interviews were completed by two interviewers, SD-S and SH (n=11, 78%). For practical reasons, three interviews were conducted by SD-S only. No-one else was present at the interviews. The majority of interviews (n=13, 93%) were face-to-face and conducted in the interviewees' place of residence (hostel or Housing First setting). One was conducted online.

On the day of the interviews, SD-S introduced herself to participants as an experienced homeless and inclusion health nurse and researcher and SH introduced herself as a researcher with a lived experience of homelessness.

SD-S and SH went through the consent process thoroughly with interviewees and provided a further Easy Read Patient Information Sheet to support the process. Consent was recorded on the online UCL REDCap system which required an e-signature. Support was given with this where necessary.

Interviewees were informed that the purpose of the interviews was to guide the development of IMPROVE, and that they would not be asked directly about their own experiences. However, interviewees were told they would not be stopped from sharing their own experiences if they chose to, and this often happened naturally.

Interviewee demographics and self-reported health status were obtained at the consent stage before the interviews started. Information requested included age, gender, current housing status, duration and number of episodes of homelessness, self-reported health status and the total number of separate self-reported physical, mental health or addiction issues experienced. Self-reported health status was assessed using a single global question,^25^ as presented in table 2.

**Table 2:** Interview demographics and self-reported health status

	Participants n=14, n (%)
Age	
18–24	2 (14)
25–34	2 (14)
35–44	3 (21)
45–54	5 (36)
55–64	0
65+	2 (14)
Gender	
Male	11 (79)
Female	3 (21)
Other	0
Self-reported health status ‘How would you describe your health overall?’	
Excellent	0
Good	1 (7)
Average	3 (21)
Poor	7 (50)
Very poor	3 (21)
Ongoing (chronic) physical health conditions	
Yes	12 (86)
No	2 (14)
How many physical health issues are you experiencing	
0	2 (14)
1	4 (28)
2	2 (14)
3	1 (7)
4	3 (21)
5	1 (7)
6	0
7	1 (7)
Average	2.5
Self-reported mental health issues	
Yes	14 (100)
No	0
Self-reported addiction issues	
Yes	13 (93)
No	1 (7)
Housing status	
Hostel	12 (86)
Housing first	1 (7)
Supported housing	1 (7)
Duration of homelessness	
Less than 1 year	1 (7)
1–5 years	5 (36)
6–10 years	4 (28)
10 years+	4 (28)

The topic guides were developed by the research team, including people with a lived experience of homelessness. The questions were designed to generate reflections from people with experience of homelessness to inform decisions about adaptations to the content of IMPROVE. Interview questions were open and explored what interviewees felt it was important for health, housing, social care or voluntary sector professionals to know about, when supporting people who are currently homeless and who have advanced ill health problems.

Interviews ranged from 25 min to 1 hour and 5 min and were audio recorded. Interviews were transcribed by an external company. No repeat interviews were conducted. The researchers debriefed together after the interviews and made written field notes.

Participants were given a £20 voucher in recognition of their involvement and time, on completion of the interview.

#### Data analysis

Transcripts were analysed by two researchers—SD-S and JB—using Braun and Clarke’s reflexive thematic analysis approach.^26^ An inductive approach to analysis was taken.^21 26^ This involved initial familiarisation with the data through reading and re-reading transcripts. Initial codes were generated independently by SD-S and JB in NVivo. These codes were discussed and refined into themes through the identification of shared patterns and meanings. These themes were then reviewed in relation to the coded data and the full dataset, and discussion with SH, BH and CS. Subsequent discussion with lived experience group members (JK, SK, PB, GD, MP) led to further refinement of themes. These themes were then named and formally defined. Reflexivity was central throughout. Reflexive practices included memo writing, discussion, and iterative engagement with the data.^27^ This supported transparency and promoted analytic depth. SD-S and JB wrote up the findings, but all authors refined the manuscript. The process was highly collaborative.

### Patient and public involvement

One of the original co-applicants for the IMPROVE project had a lived experience of homelessness. SH is a paid Research Officer working for Groundswell who has a lived experience of homelessness.

The IMPROVE project has a volunteer 5-person Lived Experience Group which is supported by Pathway, an organisation which facilitates the involvement of people experiencing homelessness in research and policy work. Pathway employs a Lived Experience Programme Manager (MP) who supports the group to engage, providing practical and emotional support. The IMPROVE Lived Experience Group meets on a bimonthly basis to contribute to the research project. For this paper they supported the development of interview questions and data analysis. The themes identified were discussed and iterated with members of the group, who are co-authors of this paper. The TIFFIN guidelines for recommended practice on approaches to the co-production of palliative and end of life care were followed when considering how best to design our process for involving people with a lived experience of homelessness in this work.^28^

This includes providing bi-monthly reflective practice provided by a psychologist with extensive experience of working with people experiencing homelessness and bi-monthly one to one meetings with a member of the research team. Group members are volunteers and receive incentive vouchers for all their engagement at a rate of £20 an hour.

A summary of the findings from the project has been provided to the interviewees themselves (where they had provided direct email addresses n=6) or to the key workers and managers of services from where the interviewees had participated (with a request to the recipients to print them out and take them to the interviewee).

## Findings

### Participants

14 people consented to be interviewed. All reported being White British. The majority of participants were male (n=11, 86%).

Demographics are presented in table 2. Over half of interviewees (n=8, 58%) had experienced homelessness for over 5 years. Only one had been homeless for less than 1 year.

The majority of interviewees (n=10, 71%) self-reported that their health status was poor or very poor. Tri morbidity, the co-occurrence of physical health, mental health and addictions issues,^29^ was common and reported by 79% of participants (n=11). The average number of physical health issues reported per person was 2.5.

### Themes

Four key themes, with associated subthemes, were identified (box 1).

Box 1 **Thematic analysis themes**

**Theme 1: The impact of homelessness on health**

- The prevalence of past and current psychological trauma in people experiencing homelessness.
- High and multiple health needs.
- Exposure to deteriorating health and deaths in homelessness settings.

**Theme 2: Structural and relational barriers to equitable healthcare**

- Access to healthcare.
- Retelling your story and communication between services.
- Poor treatment and stigmatising experiences and a learnt lack of trust in professionals and service providers.

**Theme 3: Contribution of the hostel environment to poor well-being**

- The impact of chaos on sleep and mental health.
- The perceived threat of violence and feeling unsafe.
- Inescapable exposure to substance misuse.
- Variation in staff support and skills.
- Institutionalisation and disempowerment.
- Delays in accessing appropriate housing.

**Theme 4: Interviewees’ suggestions on how health and homelessness service staff can respond to deteriorating health problems**

- Building trust and peer involvement.
- Sharing information between services.
- Promoting safety and well-being in hostels.
- Views on how deteriorating health and advanced care planning can be approached in conversations by staff.
- The need for an equitable approach to palliative and end of life care.

#### Theme 1: the impact of homelessness on health

##### The prevalence of past and current psychological trauma

Participants felt it was important for services to understand the backgrounds and experiences of people experiencing homelessness, in recognition of the profound impact past trauma had had on their lives and health. This included the experiences of being homeless, as well as the experiences preceding homelessness.

Common prior events described by participants included adverse childhood experiences, traumatic life events precipitating a personal decline, family breakdowns and discharge from prison to the streets.

P5 - So my mum’s a heroin addict and a crack addict. My dad, I don’t know what he does anymore, but he was very involved in dodgy stuff. …He got out of jail when I was born, the day I was born, he got out I think two or three days later, then went back to jail.

P8 - It makes me sad thinking about it. I was ok. I worked until I was 27. Day in, day out, two-bedroom house with two children. Lost my license to cannabis. I lost my job, my career. Just went down a spiral. A bit shit.

The experience of homelessness itself was described to be traumatic, and something participants felt practitioners did not necessarily understand.

P4 - They’ve never seen, they don’t know what it’s like being scared of where you’re going to eat next or whether someone’s going to set fire to your stuff or steal your shoes, and that is what happens when you’re asleep.

##### High and multiple health needs

Interviewees described having high health needs which were often very complex. Health needs spanned physical health, mental health and addiction. Pain and mobility issues were commonly described.

P1 - Please let me educate you on my life. I've got something to give you. Yeah. Let me give something to you. Let me show you what it feels like to have seven different conditions and 15 people involved because it’s not easy.

P2 - I know pain, believe me. I’m in a lot of pain.

Notably, most interviewees were quite open to talking about their mental health challenges. This could be seen as surprising in a one-off interview with unknown researchers, but this may reflect a wider cultural shift towards greater willingness to talk about mental health difficulties.

P2 - … my head is not in the same place as anyone else’s. The things that go through my head, I hear voices and that, so my head is not like these people…

P14 - I had a massive breakdown in 2014. Basically, I went from being a normal functioning person to being unable to do things like knock on my neighbour’s door and ask for help or phone the GP. In fact, I couldn't phone, e-mail or make contact with anybody. I just was completely cut off.

Interviewees also talked frequently about the specific health impacts of addictions on themselves and others. They broadly acknowledged the severity and potentially devastating effects of addiction and appeared to link causes and effects well.

P2 - I’ve got a wet brain, I forget all kinds through my drink, so I’ve cut down with that now.

P11 - …she had her leg cut off and before she had it cut off - I didn’t even know the girl was injecting, you know, and then all of a sudden I see her in a wheelchair with one leg…

##### Exposure to deteriorating health and deaths in homelessness settings

Interviewees felt that deteriorating health, sometimes leading to death, happened commonly in homelessness services, often at very young ages. All interviewees described knowing people who had died while homeless. Emotional reactions that were expressed about this differed from highly emotional to a rather flat sense of acceptance that this was their background reality. The interviewers felt there was an inherent sense that people knew they needed to protect themselves from the potential emotional impacts of this, and that there was limited support around this.

P7 - Well, she was a nice, girl. But I don't feel no more

P8 - Someone else died in her room. She fell asleep and was sick and they found her dead.

P13 - In 20 years, I’ve known about 12 people that have died, mostly due to drugs.

### Theme 2: structural and relational barriers to equitable healthcare

Interviewees highlighted a variety of barriers to accessing healthcare, which they felt were important to highlight to health, housing and social care professionals.

#### Access to healthcare

Access to a general practitioner (or GP—the way in which UK residents access primary care) was often described to be difficult and frustrating.

P5 - …it took them so long to get my mental health sorted because getting into a doctor when you’re homeless, it’s really difficult as well. That’s another ball ache.

P4 - Basically because I’ve been on the streets for so long, I think my doctors have written me off their books so I’ve got no way of seeing a doctor without going to the hospital and it’s a long walk.

However, some other interviewees had access to specialist GP services and acknowledged that access within these services was better than in mainstream services. This demonstrated recognition of the wider funding issues involved but also evidenced the potential value of specialist services.

P10 - I almost feel guilty that I get a doctor’s appointment the next day, or the same day. It’s like my parents they try to get doctor’s appointments they’ve got three months.

Interviewees also frequently described difficulties getting to appointments. This appeared to be due to a mix of practical issues, their own competing priorities or sometimes simply being embarrassed. Interviewees suggested overall that this resulted in reduced engagement with appointments.

P1 - You know, maybe people need to be made aware of, you know, how hard things are, without having to get to appointments. And having no phone. You got no way of getting there, and people are embarrassed any way, to go to appointments because they don’t want the questions, they haven't washed, they haven't eaten.

Interviewees also reported experiencing long delays between requesting and receiving healthcare or support, and the impact this could have. This was commonly mentioned in reference to mental health support.

P14 - …that might be another year or two or three before [the psychologist] can see you, and during then [they] will expect you to cope with what you're carrying in your head.

P2 - ….they said you were supposed to see the mental health team and that, but they haven’t been coming for a bit. I know I missed an appointment, but they stopped it, I think anyway. It’s just each to their own, isn’t it.

One interviewee also gave an example where a delay in receiving a mobility aid, and the significant impact this had on their well-being.

P1 - The wheelchair makes a big difference. Because I'm literally… I'm house bound right now, you know? So, if that would have been put in place a long time ago, when it was said it was needed, you know… I wouldn't have got to this point where now it’s become an effort to even think about going out.

Interviewees acknowledged the wider National Health Service funding context, and often perceived services to be overwhelmed. However, as a result, there was a sense they were being ‘passed around’. In some cases, delays were attributed to a total lack of services.

P1 - …they always look at it as something someone else can deal with; so… if they've got too many clients or they've got another thing going on, then they will, you know, try and sort of pass it back on to somebody else. And it’s only when they're getting prompted a lot that they will get back involved

P7 - The services just aren’t there anyway. What do you do? Imagine – what I often get told is - no, the funds, the funds or whatever it may be, they aren’t there, or whatever the excuses may be.

Overall, for many interviewees, there appeared to be a learnt sense that the likelihood of getting necessary care was low.

#### Retelling your story and communication between services

Limited or lacking communication between services, or inadequate information processing were often described. Interviewees outlined both practical and emotional impacts of lack of information sharing, including a sense of re-traumatisation in some cases.

P10 - …they scanned my brain, for like a bleed on the brain, you know, a brain haemorrhage… And then I went to the doctor’s and they said there’s no record of me being there. Very strange.

P9 - like you're telling too many people everything. And by the time you’re telling another one, you've forgotten what you've told the first person.

However, it was important to note that a desire for more information sharing was not universal. One interviewee outlined a specific stigmatising experience related to information sharing:

INT: You were stitched up? In what sense? Do you mean that they've shared information that you think hasn't been helpful?

P7 - Yes, I've got called a drug addict, and that sticks. Do you know what I mean. That’s difficult, isn't.

Another interviewee talked about having a letter with personal details sent to the wrong address in error, and the difficulties that this had caused.

#### Poor treatment and stigmatising experiences and a learnt lack of trust in professionals and service providers

Many interviewees highlighted that, even when they did receive healthcare, they often experienced poor treatment. This was usually attributed to the stigma associated with addiction. This seemed particularly relevant in hospitals where addiction was seen as inducing diagnostic overshadowing, whereby symptoms are misattributed to addiction issues ahead of other causes.

P13 - Yeah, well, when I was in the hospital. When I had the cancer. Yeah. They said “Well, that’s what you get for snorting coke. For being an addict.” Yeah. But I've never done that.

P1 - Oh, my pain has been managed terribly. Terribly because it’s always boils back to the addictions, you know. ‘Oh, but we're concerned about your addictions…’ But it comes down to it, they can't keep throwing that back in your face, you know, addiction, addiction…

There was also a feeling that in some cases professionals seemed to have ‘given up’ perhaps in the context of wider system pressures, and could not always be trusted to deliver on their promises or to be an advocate on their behalf.

P13 - So people being, you know, given a sense that something will happen, and then it not happening, I'm afraid that happens all the time, doesn't it? And it really, it really damages your trust.

P6 - …they get paid anyway. They can’t be bothered any more

P5 - The system goes ‘well he’s washed up; he’s done, so there’s nothing we can do for him.” They just kind of give up.

Although it is important to note that there were some specific examples of more positive treatment, and these were very much appreciated.

P14 - …the staff were really generous. They didn't complain that I was bed blocking.

Overall though, the negative experiences appeared to have generated an underlying mistrust and lack of belief that care would be person centred. Building back trust was seen as an important focus for reducing future barriers.

P1 - Help us to build our trust back, and it can make a big difference. It really will make a big difference.

### Theme 3: contribution of the hostel environment to poor well-being

Although not originally anticipated as a core topic for discussion, the impact of the environment on health and well-being was something that participants felt strongly should be highlighted to health, housing and social care professionals.

#### The impact of chaos on sleep and mental health

Many participants described living in a hostel as chaotic and stressful, and as detrimental to their mental health.

P12 - … squabbling happens every day. Like there’s no stopping it. Squabbling is just constant. It’s a constant thing in this madhouse.

P2 - I came in here with mental problems and now I’m going to leave, I’m getting worse because my anxiety, believe me, it’s not good.

Several participants specifically mentioned difficulty in sleeping at night due to noise made by other residents and the negative impact this had on their mental health.

P2: When you’re here and you can’t sleep, all you hear is people shouting people because they want - they’re booking spice and shit. That’s all you hear all night so you can’t sleep. One time, a dude smashed through the fence, climbed on the thing, and tried to break through and do that, like they know me. Honestly, you can’t sleep here.’

#### The perceived threat of violence and feeling unsafe

Many interviewees talked about a perceived threat of violence and other dangers being ever present throughout hostel life. This contributed to a sense of stress and foreboding which was not conducive to well-being.

P7 - Everyone’s at one another… Something bad will happen one day you mark my words.

P9 - …when things escalate. Anyone could get stabbed.

P9 - If X got sent to prison, if he couldn’t protect me. If that happened tomorrow, I'd be gone. Yeah, I don't know where. I haven't got a clue, but I would not be able to stay.’

The sense of danger was felt particularly acutely by women in mixed hostel environments. This danger was seen as being both caused and compounded by substance misuse, directly and indirectly. For instance, active disputes over drug supplies and the impact of used needles being improperly disposed of were both raised.

P7 - It’s worse than jail in a way, because you’ve got drugs and alcohol whacked into the equation.

P5 - You always see a set group of people here, a set group of people there, and then they’re all arguing over drugs and then they are fighting, end up locked up and it’s…they’re in and out of jail.

P12 - There’s needles like round the back of here. Outside this window, there’s needles. Not even with the safety caps on and everything.

#### Inescapable exposure to substance misuse

Overall hostels were seen as environments where alcohol and substance misuse occurred openly. One participant emphasised the extent to which some residents used illicit substances.

P11 - The thing is they abuse their bodies, they all take fucking like a huge amount of tablets, like Pregabs, Valium, Codeine, Xanax on top of being an alcoholic and being an heroin and crack user, yeah, and this is every day, they take copious amounts.’

Sadly, there was a sense that this was inevitable and not something that would be likely to change.

P9 - Well, it should be reported. But no one does. So you’ll know there’s someone in number 10 smoking - there’s some smoking going on in the room. Yeah, yeah. And so then they get away with it. You can hear the noise and stuff like that.

As a result, participants spoke about the risk of developing substance misuse issues, even where residents had no history of addiction before coming into a hostel, due to the prevalence of open drug use. Some relayed the anxiety they felt about this before even arriving at the hostel.

P5 - Then you can see some people come in here with no drug problems and come out with drug problems because they’ve been round it, and there’s nothing they can do about it. So then they go… someone told me if you sit in Gregg’s long enough and you don’t want a Gregg’s, eventually you’ll eat a Gregg’s, if that makes sense.

P4 - …when I found out I was coming in here, I’ve had an addictive personality in the past, and I was worried I’d start smoking spice like the rest of the lads.

In addition, nearly everyone acknowledged the difficulties of pursuing recovery and maintaining abstinence from substance misuse in this context.

P11 - But trying to get yourself clean in an environment like this when you know all these people so well, it’s hard because, you know, there’s days when I struggle, everyone struggles, you know, and I just want to go out and get high. And it would be so easy to go out my door and there’s always someone here, they’ll be like, yeah, come with me.

As a result of these impacts, one younger participant expressed concern about what the future might look like for hostel residents.

P12 - Where do you see yourself in 20 years’ time? I don’t see anyone being in here, that is proper bad… I don’t see them here in 20 years’ time. I see them in a box in the ground.

Overall, participants felt that hostels could be extremely bad for your health and well-being and were often not conducive to recovery and well-being.

#### Variation in staff support and skills

All participating hostels had support staff on site 24 hours a day. In general, the staff support in these hostels was viewed positively, but the significant challenges that staff faced were also noted.

P2 - …they do help honestly, believe me. Staff do help… L’s always on with my appointments because she reminds me because I’ll forget.

P8 - She’s got a lot of things to deal with. So, I don’t always see her when I want to see her. But yeah, currently she’s great and she will give me things.

However, staff were sometimes felt to be too ‘hands-off’, or support was noted to be variable between staff. Interviewees often seemed to recognise that they needed higher and different types of support than could be provided by hostel staff. Specific concerns about night staff being less engaged were also expressed.

P12 - …certain staff members will get done what needs to be done or help you. They’re really helpful, they do this - You know what I mean, they do everything they can for you, but other certain staff members just dib you off, you know - Do you know what I mean?

P9 - I think it’s okay. But it’s the wrong support. I haven’t got the support I need. They just go, ‘Are you all right?’ You go, ‘Yeah.’ ‘Cool,’ shut the door.

P12 - The nighttime staff are just more laid back. I've seen like - You’ll go up there and ask for a coffee or something or a hot chocolate or tea or something and there’ll just be people sat there watching something on their phones. I'm like well, that’s not doing your job. You’re meant to be alert for the 50 something people here.

There was also often a sense that both day and night hostel staff were sometimes ill-equipped to deal with the poor health of residents, or some of the issues presenting.

For example, there was a perception that staff needed more specific training relevant to the high-risk environment. One interviewee recounted an incident where they intervened when another resident was experiencing a medical emergency, noting that the support staff present had not, until then, stepped in.

P11 - I’ve come down and he’s - I’m sitting downstairs, there was people around, like the night staff, and I was like - He was just lying on his front, fitting, like frothing at his mouth. I was putting him in the recovery position and he’s quite a big lad, so I put his hand underneath head - Trying to put him in the recovery position, and staff was - Help me put him in the recovery position, why have you not done this already, I don’t know what you mean. And I just thought, you don’t even have basic first aid and you’re in a - You’re working in a night shelter.

Another issue related to appropriate infection control precautions within hostels. For example, one interviewee highlighted a perceived lack of staff awareness and communication around potentially communicable diseases among residents.

P11 - …people start getting sores in them and I’ve starte d- You know, like loads of people have it, not just in here, outside and, you know, it was - Like asking the staff what is it, is it contagious, oh we don’t know you’ll have to speak to him. Obviously, they don’t know…

Overall, interviewees recognised that staff needed significant levels of training to do their jobs within hostels.

#### Institutionalisation and disempowerment

Participants reflected on the differing levels of need among hostel residents and how staff should promote independence in line with the capabilities of each individual where possible. There was a conflict between some hostel residents needing more support, and others almost having too much. Some interviewees felt that the lack of independence, or institutionalisation experienced when living in a hostel, left many unprepared for independent living beyond the hostel:

P11 - But the kind of people like, you know, quite a few of them, they don’t even clean their rooms, staff clean their rooms for them, you know… It’s not teaching them responsibility if they’re cleaning their room every fucking, you know, couple of weeks.

Several interviewees indicated that support into employment would provide meaningful activity and purpose as part of a route to recovery and well-being. However, it was noted by some participants that this wasn’t possible when living in a hostel.

P11 - I’m a qualified barber, I want to work, I can’t work in here because you tell me that, you know, unless you’re earning really, really good money, the amount they’re charging you for a room it’s ridiculous so, they advise us not to go to work

P4 - I was working while I was on the streets and since I’ve come in here, I can’t work.

#### Delays in accessing appropriate housing

Hostels are designed to be temporary places of residence and a step on the journey to more independent living. Interviewees noted a contradiction between this aim and the length of time many people had remained in hostels.

P7 - They said it should be couple of months, 2-3 months. I mean, that would be reasonable. But people have been too long, people have been here years. One person has been here 8 years.’

Overall long delays for people moving on from hostels seemed to have led to a despondency about the future, and long periods in hostels were particularly felt to be inappropriate for people with physical health issues:

P2 - It’s like with T, I think he’s a poorly man, but he’s already been here over two years, not good considering he’s not well.

### Theme 4: interviewees’ suggestions on how health and homelessness service staff could respond to deteriorating health problems

#### Building trust and peer involvement

In general, interviewees indicated that one of the biggest enablers of better care was a more personalised approach to care, with a focus on understanding each individual and enabling the building of trust.

P1 - Well, they need to learn about the person’s background, and you know, understand it. They need to know what they can do to, you know, help that person, where the problem stems from, you know.

P13 - they need to listen to people and really listen to them. Not something like all the intellectual stuff.

Many people saw listening to people with lived experience when designing services and/or employing peers within services as being key to this.

P4 - Yeah. You can’t really understand it until you’ve lived it yourself.

P13 - If you're going to learn about it, you have to listen to those people that have been there.

Several interviewees also suggested that staff shadowing a person experiencing homelessness would be the best education a practitioner could get in terms of challenging internal bias or discriminatory attitudes to promote compassionate care. Shadowing within homelessness services was also felt to be a realistic and reasonable route to gaining insight into challenges, experiences and opportunities.

P1 - Maybe, go out and follow somebody that’s homeless. Everyone should follow somebody that does have addiction, to make them see how hard everyday life is. You know, we don't get up in the morning and have a shower, go to work, come home, cook. No, we don't have that.

#### Sharing information between services

In many cases, interviewees wanted more communication between different professionals and across services like health and housing providers to enable better, more joined-up care.

P9 - They all need to be on the same page. They all need to communicate… I mean without that, like you're telling too many people everything.

P12 - Yeah, it’s easier for them to do it. [Share information] So, then it all like gets done quicker and I don’t have to go feed it to them and then go back to them again…

In contrast, other interviewees remained unsure about the sharing of information due to their past experiences and mistrust of services.

P7 - No, they should do their jobs separately and properly. End of story.

#### Promoting safety and well-being in hostels

Interviewees talked about how staff could improve safety in hostels. This included several references to staff conducting more room checks.

P11 - They should pull them up and tell them, you know, I’m putting on extra welfare checks. There shouldn’t have to be an incident for them to be like oh, he’s OD’d, so now we’re going to put him on extra welfare checks, you know.

There were also suggestions for less tolerance of open substance use in temporary accommodation.

P9 - I think there could be a bit more tightening on the reins, you know, realistically, on what actually goes on.

Training around the management of medical emergencies was also felt to be important, and harm reduction approaches were mentioned, although with caveats.

P11 - They should be aware and should know basic first aid, you know, working somewhere like this.

P11 - They give a resident in here a SWAP [Safer Works Access Programme] pack then let them back into their room straightaway, and they OD in their room.

Finally, it was suggested that vulnerable people without addictions should not really be placed in a hostel at all, indicating a need for a range of different housing options reflecting that range of experiences that fall under the umbrella of homelessness.

P12: I reckon they should move like the ones who haven’t really got habits or anything.

#### Views on how deteriorating health and advanced care planning can be approached in conversations by staff

Interviewees shared their perspectives on how professionals could approach conversations about deteriorating health and end of life topics with service users.

Exploring people’s wishes and preferences were perceived as important and relevant issues, which would benefit from a proactive approach. However, it was also recognised that these could be difficult conversations to have, given the level of trauma and exposure to death and dying among service users.

P10 - Yeah, yeah, but I guess it’s a sensitive issue isn’t it. It’s like they feel that they - You know, that they don’t want to add to the problem, but I guess if you sort of read the character of what they’re like you can maybe - Or just slowly go into it - You know, just sort of suggest that maybe slow down a bit or there’s dangerous stuff around at the moment or do you want to live, you know, it’s like - You know, just get someone to talk to them.

Interviewees felt the way professionals approached these conversations was important and a trusting relationship was an important pre-requisite for exploring issues related to the future and deteriorating health.

P13 - You need to have the conversation with someone trusted, the key worker. Yeah. And if someone that’s saying all that to you, that’s not your key worker or someone you don't trust, you're not going to listen to them or talk to them. Yeah. So if it comes from someone that we trust in, then you'll tell them, you know, this is what I want. And could you help me with this and like, can you help me with this too?

Some interviewees wanted hostel staff to intervene more actively, particularly in cases where residents seemed at greater risk of harm or deterioration in health.

P9 - They need to take it on their own initiative to go and get the relevant people or something and come and try and talk to this person before it’s too late. Whether the person receiving this wants it or not.

However, one interviewee noted that given the high level of ill health and risk experienced by many people in temporary accommodation, conversations about the potential risk of dying were so common that they no longer had an impact on residents. Therefore, a different type of conversation was needed to make an impact, perhaps one that outlined choices, rather than focusing on consequences.

P11 - That might be the kick up the arse they need as well if they’re not - You know, they may take stuff serious if they’re that close. Like, all of us hear it, so many times ‘you’re going to kill yourself, you’re going to die, you’re going to end up dead, you’re going to end up the same as so and so’… but it isn’t heard.

Generally, interviewees felt key working conversations and future planning conversations involving next of kin and key family and friends were appropriate and an important part of someone’s story. Interviewees’ thoughts seemed to naturally turn to their families when talking about deteriorating health. This was interesting, as there is a tendency in the sector to think that most people have few family connections.

P2 - It’s just my kids are the most important, that’s what I know I keep mentioning, my kids, that’s the only thing that’s well in my life.

Interviewees talked freely about family issues, changing family dynamics and their regrets – without prompting on this topic. They noted that key contacts could change and might need regular updating. Others mentioned that, for people who do not have friends or family to act as next of kin, a support worker could fulfil this role.

INT: So do you think people should be asked again about those things, aboutwho’s important to them and who their next of kin is?

P4 - I do think that, yeah … families fall out all the time.

P5 - obviously key workers are like your next of kin when you’re homeless because you haven’t got many people.

#### The need for an equitable approach to palliative and end of life care

A small number of interviewees offered their thoughts on end-of-life care specifically, when this developed naturally as part of the conversation. Thoughts generally focused on comfort and wanting to be treated equitably.

P1 - They can't seem to, but they do have that duty to make you comfortable. And it’s just a small price to pay back. I understand they can't do it with everybody. But if there are ways round the problems, you know, just to make that person feel a bit more comfortable.

P14 - So, I'm really saying give people the right and the dignity that we'd expect for everyone as they approach to the point where there may be no return.

Similar to the other areas discussed, there was a powerful sense that care should be truly person-centred, given the histories of trauma and negative experiences that many people experiencing homelessness have endured.

## Discussion

Overall, this small study has uncovered key insights about the level of exposure to deaths by people experiencing homelessness, a lack of access to mental health support when someone has high physical health needs, high levels of stigma and service mistrust, concerns about safety and well-being in hostels, and the need for more proactive conversations about deteriorating health. Interviewees described a high burden of health issues and past psychological trauma, echoing previous research with this population.^1 4 29^ However, the extent to which interviewees articulated a sense of ongoing closeness to deaths among their peers was striking and underlined the actual high level of deaths in hostels. This situation seemed to be adding further trauma to past trauma. Song *et al*^30^ and Purkey and MacKenzie^31^ noted similar findings. Although the psychological effects of homelessness itself are well known,^32^ the specific impact of living alongside high levels of death appears less well researched. This finding has implications for research and practice relating to bereavement support for temporary accommodation residents.

Many barriers to and within primary and secondary healthcare described in this paper reflect previous research.^1 3–6^ A striking finding from this work was the evidence of long delays in access to over-subscribed services, or services that were simply absent. Such issues also affect many people in the general population,^33^ but the consequences of this for people experiencing homelessness are compounded by issues such as frequent relocation. Drawing on NICE guidance Torjesen^34^ suggests people experiencing homelessness should be prioritised on waiting lists because of the elevated need in this population.

Barriers to mental health services were commonly described, a finding consistent with other recent reports.^6 35 36^ However, interviewees talked freely about their mental health and demonstrated a clear desire to engage with mental health services. In fact, they often suggested that mental health difficulties underpinned the many factors associated with their deteriorating health. This represents a key issue for commissioners, including considering mental health provision within palliative and end of care services, which may need to be adapted to support people experiencing complex trauma.

Interviewees emphasised that addressing stigma and discrimination, alongside the provision of personalised care and peer support, is central to rebuilding trust in services. Addictions were frequently identified as a key driver of stigma, particularly in the context of pain management, a finding consistent with previous research.^37^ Given the high prevalence of substance use issues among people experiencing homelessness, these factors require careful consideration across all healthcare services, including specialist palliative care.^38^

The hostels in this study were perceived as being often dangerous and bad for mental and physical well-being due to exposure to violence, substance misuse, poor infection control and under-supported staff. Four people interviewed for this study were also under 30, which is highly concerning and highlights the importance of further investigation to determine whether this finding is more widely applicable.

The suggestion that hostels are not conducive to well-being is not new^39–42^ and the fact that this concern persists is perhaps understandable in the context of average 40% Local Authority budget cuts across England.^43 44^ However, evidence indicates that hostel residents may be experiencing worse health outcomes and increased mortality over time.^45^ These findings are particularly troubling when considered alongside interviewees’ concerns about lack of move-on to more settled accommodation. There appears to be a clear role for safeguarding and death reviews to understand the impacts of these funding cuts on care provision. Cuts to young people’s homelessness services also seem particularly inappropriate in this context and should perhaps be challenged.^46 47^

Interviewees recognised that health often deteriorates when someone is in a homelessness service and that sometimes conversations about this were not being had. The findings of this study support previous research highlighting the need for an approach to supporting people experiencing homelessness with deteriorating health which incorporates uncertainty and promotes well-being, dignity and choice.^19^ It was interesting to note that many interviewees had an interventionist stance, particularly in the case of addictions. In fact, there is plenty of evidence that most people want active help, if not always in the way it is presented.^48 49^

Exploring connections with family and friends in relation to deteriorating health was also seen to be very important. Interviewees spoke freely about family situations without being asked about this. The issues explored in the interviews, such as support for people with deteriorating health, and deaths among peers may have prompted interviewees to reflect on their own family situations or valued connections. These discussions did not appear to cause distress and interviews described how family connections were subject to change. Taken together, a potential need for more regular discussion about nominated next of kin and significant personal relationships may be required.

### Practical use of the interview findings and the value of co-production

The interview findings were discussed with the IMPROVE Lived Experience Group and informed the narrative and focus of 18 short films in the updated IMPROVE programme. 10 of the films feature people with lived experience of homelessness, 4 feature only people with lived experience. Topics explored in the films include access to care, the impact of trauma, personalised care, the myth of lifestyle choice, challenges in pain management and bereavement support, many of which were key themes from these interviews. In addition, IMPROVE focuses on capability and capacity building in hostel staff with regards to issues identified in this research. Shadowing has also been recommended throughout the programme in response to interviewees’ recommendations.

This work builds on prior work of the research team in developing the TIFFIN recommendations^28 50^ and also other coproduced staff training initiatives in homelessness,^51 52^ demonstrating the value of co-producing the resources for a range of professionals.

### Implications for practice

- Interviewees described high levels of exposure to death and dying. Consideration should be given to the types of bereavement support that could be offered in recognition of the impact of these experiences.
- Services, including palliative care services, should be aware of the role of trauma and addiction in the journeys of people experiencing homelessness and adopt a non-stigmatising and person-centred approach to support.
- Commissioners and service providers should consider the level of exposure to violence and drug use and the impact of chaotic hostel environments on poor sleep. Consideration should be given to potential changes to supported accommodation pathways to mitigate these issues.
- When handled sensitively, conversations about what is important to people with regards to their health and how they would like to be cared for can be beneficial as health deteriorates. Homelessness services may need to think about the training that staff may need to support this.
- This work demonstrates the potential future benefits of co-producing training, quality improvement or Community of Practice initiatives.

### Strengths and limitations

The number of participating services and service users is relatively small and men and White British people are overrepresented. All participants were self-selecting which could have introduced a bias in the sample. As such the results did not capture the experiences of people from a diverse range of ethnic backgrounds, particularly those with no eligibility for housing support in the UK. With regard to women, the smaller numbers do reflect the common gender balance seen in many homelessness services. Women have been found to be more likely to be experiencing ‘hidden homelessness’ than be in the hostel systems.^53^

All three participating hostels were large-scale settings. There has been a shift towards reducing the size of hostels in recent years to combat some of the above issues.^54^ However, while these contextual factors are important, they do not negate the concerns of interviewees.

The involvement of people with lived experience across all stages of the study brings strength to the findings. These data were collected as part of a larger project and so had a direct route to impact, via informing the shape and content of IMPROVE, an intervention designed to support local, interprofessional working around palliative care and homelessness.

#### Suggestions for further research

This work points to important areas for future research including the emotional and psychological effects of sustained exposure to death among people experiencing homelessness, the need for bereavement support and the implications of temporary accommodation for health and well-being. There is also a need for a larger sample that recruits a more diverse sample in terms of gender, ethnicity and geography.

## Conclusion

Deteriorating health leading to death among people experiencing homelessness was widely recognised by interviewees, who felt that sensitive conversations about preferences for future care issues could be initiated by staff.

However, interviewees also emphasised that such discussions should be accompanied by opportunities to live in healthier environments, rather than settings that may introduce or sustain risks to well-being. Improved access to health and care support is also needed to reduce the inherent risks of dying.

## Data Availability

Data are available upon reasonable request.
