# Supplementary material for: Exploring the views of people experiencing homelessness regarding their health and housing support, and their perspectives on deteriorating health and end of life support: a qualitative analysis
Source: BMJ Open. 2026 Oct 9;16(10):e121183. doi: 10.1136/bmjopen-2026-121183 (PMC13656098; doi:10.1136/bmjopen-2026-121183)
Supplement: Supplementary file 1 [file bmjopen-2026-121183.reviewer_comments.pdf]

PEER REVIEW HISTORY

BMJ Open publishes all reviews undertaken for accepted manuscripts. Reviewers are asked to complete a checklist review form and are provided with free text boxes to elaborate on their assessment. These free text comments are reproduced below.

ARTICLE DETAILS

Title (Provisional)

Exploring the views of people experiencing homelessness regarding their health and housing support, and their perspectives on deteriorating health and end of life support: a qualitative analysis

Authors

Dorney-Smith, Samantha; Barnett, Joel; Hough, Sarah; Kennedy, Joanne; Khan, Sahar; Bull, Pete; Davis, Gareth; Pattinson, Mandy; Shulman, Caroline; Hudson, Briony

---

VERSION 1 - REVIEW

**Reviewer**	**1**
**Name**	**Robinson, Jackie**
**Affiliation**	**University Of Auckland**
**Date**	**20-May-2026**
**COI**	**None**

This is a really important topic and addresses a number of issues related to appropriate care at the end of life for people who are experiencing structural vulnerability. Foregrounding lived experience is a real strength to this study and provides valuable insight into the reality of people who are traditionally underserved by health and palliative care services. I hope the authors find this feedback helpful.

Introduction would benefit from a more robust discussion of the rationale for this study. The first paragraph begins to do this but requires more detail in regards to how palliative care currently does not meet the needs of this population. More detail on how health and palliative care is traditionally provided in the UK and how this model may not currently meet the needs of the homeless population would also be useful.

The second and third paragraph where the larger study is introduced would fit better in the methodology section. More detail is needed to explain what IMPROVE is and how it has been developed and its relationships with this current study i.e. was IMPROVE an output from the study or was it undertaken in parallel?

Last sentence of the introduction needs to clearly state the aim of the study. The way it is currently written is not really stated as an aim or objective. Also, what is meant by the "inclusion health sector".

Methodology - more detail needed here. What was the philosophical framework used to interpret the data and why? More detail on how participants were identified and recruited for the study is needed. For example, what was the eligibility criteria and who applied the criteria to potential participants?

What is the relevance of "33 practitioners" being interviewed to this study - perhaps this detail could be removed? The methodology section would benefit from application of the Consolidated Criteria for Reporting Qualitative Research (COREQ) as a framework to ensure all the relevant detail is included along with justification for the chosen theory, approach and method.

How was the sample size decided? What was the role of approaches such as data saturation or information power in deciding on the sample size?

Researcher positioning - good to see this included as it is important when interpreting the findings. What is NIHR research protocol mean? International readers may not understand these abbreviations.

Interviews: what was the rationale for using semi structured interviewing? How was the interview guide developed? Who decided on the questions being asked and how were they related to IMPROVE exactly? Where did the face-to-face interviews take place?

What is meant by "personal experiences" in the context of asking participants about their lived experiences? Needs a bit more explanation of this approach as it seems quite important in terms of the questions asked of participants. I am not sure I quite understand the difference between "lived experience" and "personal experience". Perhaps examples of what is meant by ‘personal experience’ would help here.

Findings - reference is missing in the second paragraph. Was a specific tool used for participants to rate their health status? How was this applied during the interview?

Aims are clearly stated but I would like to see an overall aim stated at the end of the introduction which is supported by the rationale for the study.

Research participants and settings - please explain what "Local Authority" meands and describe what a "housing First" placement is for international readers. What support did hostel workers provider in relation to recruitment exactly? What sampling approach (beyond purposeful sampling) was used for the remaining 12 participants? And why was this approach used? How might it have impacted on the findings?

Please review the sentence "Interviews were taken transcribed by an external company"

Data analysis: What was the rationale for using reflexive thematic analysis? What is meant by "candidate themes". You state that "reflexivity was central throughout" - how was this achieved exactly? Was an inductive or deductive approach used in the analysis of data?

What is Groundswell? Is Hostel living in the UK temporary housing?

Findings: there are a lot of sub themes of which many appear to be interconnected and perhaps could be refined/combined to provide a more rich in depth interpretation of the data. As it currently stands, the findings feel somewhat descriptive and it is difficult to follow the analytic narrative. For example, integrating an interpretation of the ‘how and why’ to

participant responses would provide a more enriched exploration of what it means to be experiencing homelessness while living with mental health and addiction issues as well as poor health. There are also too many quotes used throughout which makes the interpretation fragmented. Usually, one quote for each point being made is adequate.

Please review what appears to be an unfinished sentence: “There was often a sense that both day and night hostel staff were sometimes ill equipped to deal with the poor health of residents, or some of the issues presenting h” (page 16)

Discussion – what are the key findings from this study that adds to the current evidence. State this clearly and at the start of the discussion.

Interpreting the key findings is important and needs to be integrated throughout the discussion. For example, an interesting finding is highlighted as “ the extent to which interviewees articulated a sense of closeness to the deaths of their peers was striking” – why do you think this was? How can you use the wider literature to support your interpretation of this finding?

Another example is the finding on barriers to accessing health care which is well known as the authors say. But why do you think participants are stigmatised when they engage with health services? What does this say about healthcare professionals practice and the attitude of the general population towards people experiencing homelessness. Bring in the wider sociology literature to support your arguments about the how and the why to the findings.

It is interesting that all the participants were White British. How might this have impacted on the findings given the high number of migrants who experience structural vulnerability globally?

All participants reported issues with mental health. How might this impact on how palliative care is provided? Similarly, all but one had issues with addiction. These are important findings to explore further in the discussion.

Reference list needs revision to meet the requirements of the journal especially when referencing websites.

**Reviewer**	**2**
**Name**	**Mattsson, Elisabet**
**Affiliation**	**Ersta Sköndal Bräcke University College**
**Date**	**02-Jun-2026**
**COI**	**None**

Please find below my comments on the manuscript *Exploring the views of people experiencing homelessness regarding their health and housing support, and their perspectives on deteriorating health and end of life support* (bmjopen-2026-121183).

This is an important and timely qualitative study exploring the views of people experiencing homelessness regarding health, housing, and end-of-life issues. The focus

on an under-researched area and the involvement of co-researchers with lived experience are clear strengths. The data are rich and provide valuable insights with relevance for practice and policy.

However, the manuscript would benefit from strengthening in five key areas: 1) methodological transparency and reflexivity; 2) clarity around co-production and the analytic process; 3) analytical depth and alignment with the stated qualitative approach; 4) coherence and integration in the discussion; and 5) more explicit engagement with relevant literature and more precise referencing.

Methodological considerations

Sampling and gender representation

The sample appears to be predominantly male, with most participants recruited from male-only hostels, resulting in three female and eleven male participants. Given the well-established gender dimensions of homelessness, particularly in relation to safety, trauma, and service access, this imbalance should be explicitly acknowledged as a limitation. It also has implications for interpretation, as the findings may disproportionately reflect male experiences of homelessness. This needs to be addressed in the discussion section.

Reflexivity and interviewer positionality

The involvement of a co-researcher with lived experience of homelessness in data collection is a significant strength and adds important experiential expertise to the study. However, the manuscript would benefit from a more explicit reflexive discussion of how positionality may have influenced the research process and the construction of the findings.

Beyond issues of sample composition, the interviewer-participant relationship also warrants further consideration. The interviewers (a researcher and a co-researcher with lived experience) appear to be female, while most participants were men. This creates a complex social dynamic that may have influenced trust and rapport, disclosure of sensitive experiences, and the ways in which topics such as health, trauma, violence, and substance use were discussed during the interviews. It may also have shaped how participants chose to present their experiences and how particular issues were explored in the interview setting.

Given the study's emphasis on lived experience involvement and co-production, a more explicit reflection on these relational dynamics would strengthen methodological transparency and provide greater insight into how knowledge was co-produced throughout the research process.

Analytical approach and co-production

While the study is presented as using reflexive thematic analysis, several themes appear primarily descriptive and topic-oriented rather than organised around broader interpretive concepts. The findings are richly illustrated with participant quotations, but the analytic narrative remains relatively limited. The manuscript would benefit from making the interpretive work of the researchers more visible, including how patterns of shared meaning were developed and how themes move beyond topical categorisation toward more conceptual insights.

Furthermore, the manuscript highlights co-production and lived experience as central strengths. However, it remains unclear how these were operationalised in practice throughout the study. While reflexivity and author contributions are described, the relationship between these sections is not fully coherent. It is unclear:

- How contributors with lived experience were involved in coding, theme and sub-theme development, and interpretation
- How analytic decisions were made
- How disagreements or alternative interpretations were handled
- How contributions were integrated into the writing process

In addition, greater detail regarding the lived experience steering group's involvement would be valuable. For example, information about the frequency and duration of meetings, the stages of the study at which the group was involved, and the nature of their contributions to analysis, interpretation, and manuscript development would help readers better understand the extent and influence of the co-production process.

The manuscript would also benefit from greater transparency regarding practical aspects of lived experience involvement, including whether members of the lived experience group received financial remuneration for their contributions, the basis on which this was provided, and whether any formal employment or consultancy arrangements were in place.
Greater transparency regarding who was involved at different stages of the analysis, how the analytic process evolved, and how decisions were reached would strengthen the credibility and rigour of the study.

Results

The results section is clearly structured and presents rich qualitative data. However, quotations at times appear to carry much of the interpretive burden. Additional analytic commentary would help clarify how the authors moved from individual accounts to broader patterns of shared meaning.

Discussion

Need for a more integrated analytical narrative

The discussion closely follows the structure of the results, addressing each theme in turn. While this approach is common, it leads to a fragmented reading experience and limits the development of a cohesive interpretive argument. The discussion would benefit from identifying a smaller number of overarching interpretive insights that cut across themes, rather than discussing each theme largely in isolation.

Linking methodological limitations to interpretation

The discussion would also benefit from more explicitly linking methodological issues (e.g. gender imbalance, interview dynamics, descriptive analysis) to the interpretation of findings. This would increase analytical transparency, clarify the scope and limits of the conclusions, and strengthen the credibility of the study.

Literature and referencing

Engagement with PPI and co-production literature

Given the central role of lived experience in the study design and interpretation, there is limited engagement with the Patient and Public Involvement (PPI) and co-production literature. Stronger theoretical positioning in relation to this body of work would contextualise the methodological approach, highlight the study's contribution, and strengthen its conceptual grounding.

Precision and alignment of citations

There are instances where references appear broad or not fully aligned with the claims being made. Improving the precision of citations and ensuring clear alignment between claims and supporting evidence would strengthen the manuscript's rigour.

Contribution and implications

The study offers important insights into the lived realities of people experiencing homelessness, particularly in relation to deteriorating health, exposure to death, and barriers to care. The emphasis on trust, person-centred approaches, and lived experience is highly relevant. Addressing the issues outlined above would significantly enhance the analytical coherence, methodological transparency, and overall impact of the study.

Recommendations

- Strengthen methodological transparency regarding reflexive thematic analysis, reflexivity, and co-production.
- Reflect more explicitly on researcher positionality and the implications of

the gender imbalance in the sample.

- Develop a more interpretive and conceptually integrated analysis.
- Reorganise the discussion to provide a clearer overarching analytical narrative.
- Link methodological limitations more explicitly to the interpretation of findings.
- Strengthen engagement with relevant PPI and co-production literature.
- Improve the precision of referencing and alignment between claims and supporting evidence.

VERSION 1 - AUTHOR RESPONSE

**Reviewer: 1 Dr. Jackie Robinson, University Of Auckland Comments to the Author:** This is a really important topic and addresses a number of issues related to appropriate care at the end of life for people who are experiencing structural vulnerability. Foregrounding lived experience is a real strength to this study and provides valuable insight into the reality of people who are traditionally underserved by health and palliative care services. I hope the authors find this feedback helpful.	
Introduction would benefit from a more robust discussion of the rationale for this study. The first paragraph begins to do this but requires more detail in regards to how palliative care currently does not meet the needs of this population. More detail on how health and palliative care is traditionally provided in the UK and how this model may not currently meet the needs of the homeless population would also be useful.	We have added further detail on this in the introduction. More could of course be added, but we feel this is the right amount for the length of this article.
The second and third paragraph where the larger study is introduced would fit	The methods section has now been expanded to include the information that was previously in the introduction section, and the links to IMRPOVE have been expanded upon.

better in the methodology section. More detail is needed to explain what IMPROVE is and how it has been developed and its relationships with this current study i.e. was IMPROVE an output from the study or was it undertaken in parallel?	
Last sentence of the introduction needs to clearly state the aim of the study. The way it is currently written is not really stated as an aim or objective.	The introduction has been thoroughly revised.
Also, what is meant by the "inclusion health sector".	NHS England defines inclusion health as<br><br>*"an umbrella term used to describe people who are socially excluded, who typically experience multiple overlapping risk factors for poor health, such as poverty, violence and complex trauma. This includes people who experience homelessness, drug and alcohol dependence, vulnerable migrants, Gypsy, Roma and Traveller communities, sex workers, people in contact with the justice system and victims of modern slavery."*<br><br>We have edited the sentence in the introduction in which this appeared for clarity.<br><br>We have also added clarity to the meaning of the role of SDS who is a homeless and inclusion health nurse.
Methodology - more detail needed here. What was the philosophical framework used to interpret the data and why?	The following has been added to clarify the philosophical framework we have applied:<br><br>*"An interpretivist approach guided this study, recognising that individuals interpret and make meaning of their experiences in diverse ways (Pervin, 2022). Our analysis aimed to understand how participants constructed meaning around questions related to the health and support for people with declining health in the context of homelessness, rather than to identify a single underlying reality."*

More detail on how participants were identified and recruited for the study is needed. For example, what was the eligibility criteria and who applied the criteria to potential participants?	Eligibility criteria has now been added to the methods section. We have also expanded the recruitment section to provide more information about how participants were identified, screened and approached.
What is the relevance of "33 practitioners" being interviewed to this study - perhaps this detail could be removed?	This was included to give more information about the wider IMPROVE evaluation, of which this analysis is a part.
The methodology section would benefit from application of the Consolidated Criteria for Reporting Qualitative Research (COREQ) as a framework to ensure all the relevant detail is included along with justification for the chosen theory, approach and method.	Thank you, we have now completed the COREQ and added the additional detail required to the manuscript.
How was the sample size decided? What was the role of approaches such as data saturation or information power in deciding on the sample size?	The Reflexive Thematic Analysis Reporting Guidelines (RTARG) advise against using data saturation as a means of determining sample size.<br><br>Saturation assumes that themes are "within" the data and can therefore be exhaustively identified. Reflexive thematic analysis conceptualises themes as interpretive and actively constructed by the researcher. Therefore, sample size in this project was guided by the aim of generating rich, meaningful data to address the research question. Practical considerations also came into play as we wanted to ensure that our researcher with lived experience was able to play a role in as many interviews as possible.

Researcher positioning - good to see this included as it is important when interpreting the findings. What is NIHR research protocol mean? International readers may not understand these abbreviations.	The NIHR protocol referred to in the manuscript is the protocol for the IMPROVE project. We have changed the wording of this section for clarity.
Interviews: what was the rationale for using semi structured interviewing? How was the interview guide developed? Who decided on the questions being asked and how were they related to IMPROVE exactly?	We have expanded the data collection section of the methods section to clarify these points.
Where did the face-to-face interviews take place?	The majority of interviews took place within the services that participants were residing. One out of the 14 interviews took place online. This has been added to the methods section.
What is meant by "personal experiences" in the context of asking participants about their lived experiences? Needs a bit more explanation of this approach as it seems quite important in terms of the questions asked of participants. I am not sure I quite understand the	Personal experiences relate to lived experience. For clarity we have used the term lived experience, rather than personal experience throughout the paper.

difference between "lived experience" and "personal experience". Perhaps examples of what is meant by ‘personal experience’ would help here.	
Findings - reference is missing in the second paragraph.	Thankyou, this has now been added.
Was a specific tool used for participants to rate their health status? How was this applied during the interview?	A single item measure was used to assess self-reported health status. The question, which is commonly used in health research (Desalvo et al, 2006) was “overall, how would you rate your health?” Excellent/ good/ average/ poor/ very poor.<br>This information was collected as part of the demographic questions collected during the consenting process.<br><br>Information to clarify this has been added to the methods section.
Aims are clearly stated but I would like to see an overall aim stated at the end of the introduction which is supported by the rationale for the study.	The introduction has now been re- written and an aim added at the end of this section
Research participants and settings - please explain what "Local Authority" means and describe what a "Housing First" placement is for international readers.	In the UK a “Local Authority” is the part of government that runs local services in a specific area (e.g. a city, borough, or county), rather than at national level.<br><br>Housing First placements provide permanent, self-contained housing with no preconditions, alongside flexible support. In contrast, homeless hostels offer temporary, where access and continued stay are typically contingent on meeting behavioural or engagement requirements.<br><br>Both are now explained in the text.
What support did hostel workers	Hostel workers shared information about the study with service users via posters and in key work sessions, and provided verbal information and a Participant Information Sheet in advance to

provider in relation to recruitment exactly?	people that said they might be interested in participating. More detail relating to this has been added to the manuscript.
What sampling approach (beyond purposeful sampling) was used for the remaining 12 participants? And why was this approach used? How might it have impacted on the findings?	Hostel workers were asked to approach people with high health needs that they thought might be interested in participating in the two weeks running up to the interview day. The hostel workers provided verbal information and a Participant Information Sheet in advance to people that said they might be interested in participating. They then kept a list of interested people to target on the day.
Please review the sentence "Interviews were taken transcribed by an external company"	This has been reworded as follows *"interviews were transcribed by an external company"*
Data analysis: What was the rationale for using reflexive thematic analysis?<br><br>What is meant by "candidate themes".<br><br>You state that "reflexivity was central throughout" - how was this achieved exactly?<br><br>Was an inductive or deductive approach used in the analysis of data?	Reflexive thematic analysis was selected for its flexibility and suitability for interpretive qualitative research, enabling in-depth exploration of patterns of meaning across interview data while recognising the researcher's active role in knowledge production.<br><br>Candidate themes are initial, evolving groupings of patterned meaning across the data that are identified during analysis but have not yet been finalised, refined, or fully defined.<br><br>Reflexivity in RTA is embedded throughout the process by recognising that analysis is an interpretive act shaped by the researcher, and by critically reflecting on that influence at every stage. Reflexive practices, including memo writing, discussion and iterative engagement with the data, supported transparency and analytic depth.<br><br>An inductive approach to analysis was taken.
What is Groundswell? Is Hostel living in the UK temporary housing?	Groundswell is an organisation which aims to raise the voice of people with a lived experience of homelessness. One aspect of this is supporting people with lived experience of homelessness to be involved and embedded into a range of research projects.

	Most hostels have a maximum length of stay and so could be considered temporary.
Findings: there are a lot of sub themes of which many appear to be interconnected and perhaps could be refined/combined to provide a more rich in depth interpretation of the data. As it currently stands, the findings feel somewhat descriptive and it is difficult to follow the analytic narrative. For example, integrating an interpretation of the 'how and why' to participant responses would provide a more enriched exploration of what it means to be experiencing homelessness while living with mental health and addiction issues as well as poor health.	We have reduced the number of sub-themes, and have increased the amount of analysis in the findings section.<br><br>The questions we were asking were not specifically not about in-depth individual experiences of homelessness and living with mental health and addiction issues – the questions were about what the interviewees thought that staff should know.
There are also too many quotes used throughout which makes the interpretation fragmented. Usually, one quote for each point being made is adequate.	We have removed several of the quotes.
Please review what appears to be an	This has been addressed.

unfinished sentence: "There was often a sense that both day and night hostel staff were sometimes ill equipped to deal with the poor health of residents, or some of the issues presenting h" (page 16)	
**Discussion**<br>What are the key findings from this study that adds to the current evidence. State this clearly and at the start of the discussion.	We have added a statement of the main findings at the beginning of the discussion.
Interpreting the key findings is important and needs to be integrated throughout the discussion. For example, an interesting finding is highlighted as "the extent to which interviewees articulated a sense of closeness to the deaths of their peers was striking" – why do you think this was? How can you use the wider literature to support your interpretation of this finding?	We have included greater interpretation of the findings, and have included some suggestions for future practice.<br><br>In respect to the closeness to deaths specifically, we believe this was a real effect of living in environments where there are multiple deaths.
Another example is the finding on barriers to accessing health care	We think the stigma is largely related to addiction and have explored that more.

which is well known as the authors say. But why do you think participants are stigmatised when they engage with health services? What does this say about healthcare professionals practice and the attitude of the general population towards people experiencing homelessness. Bring in the wider sociology literature to support your arguments about the how and the why to the findings.	Additional content and references have been added to the discussion section. In order to keep the length of the manuscript manageable, we have not included wider sociology literature.
It is interesting that all the participants were White British. How might this have impacted on the findings given the high number of migrants who experience structural vulnerability globally?	This is a good point. It was by chance that we ended up with all White British interviewees. This is not true of services in general. Depending on the area of the country between 5 and around 30% of the population are non-White British. Perhaps in a future we could purposely sample.<br><br>One element that will have fed into this is the eligibility issue. Some people who are not White British will not be eligible support, although many will be.<br><br>Further detail has been added to the manuscript including the following:<br><br>*'All the placements mentioned above require eligibility for welfare support in the UK.'*<br><br>*'All reported being White British. This was a chance finding, but may be linked to the fact that less people who are non White British are eligible for Local Authority funded support in the UK.'*
All participants reported issues with mental health. How	We have acknowledged this as a point in the discussion section.

might this impact on how palliative care is provided?	
Similarly, all but one had issues with addiction. These are important findings to explore further in the discussion.	We have acknowledged this in the discussion section.
Reference list needs revision to meet the requirements of the journal especially when referencing websites.	We have updated the reference list.
**Reviewer: 2 Dr. Elisabet Mattsson, Ersta Sköndal Bräcke University College Comments to the Author:**	
**Methodological considerations**<br><br>Sampling and gender representation The sample appears to be predominantly male, with most participants recruited from male-only hostels, resulting in three female and eleven male participants. Given the well-established gender dimensions of homelessness, particularly in relation to safety, trauma, and service access, this imbalance should be explicitly acknowledged as a limitation. It also has implications for interpretation, as the findings may disproportionately reflect male	There is a gender imbalance in visible UK homelessness with men more represented than women, and currently with many more men than women living in hostels.<br><br>*'There were also more men (11) than women (3) interviewed, although this does reflect the common gender balance seen in many homelessness services.'*

experiences of homelessness. This needs to be addressed in the discussion section.	
**Reflexivity and interviewer positionality** The involvement of a co-researcher with lived experience of homelessness in data collection is a significant strength and adds important experiential expertise to the study. However, the manuscript would benefit from a more explicit reflexive discussion of how positionality may have influenced the research process and the construction of the findings.	SDS is a female Clinical Research Associate and homeless and inclusion health nurse with 24 years' experience of working directly with people experiencing homelessness and other excluded groups. Inclusion health nurses work to support socially disadvantaged individuals to access the health care they need. SH is a female Research Officer for Groundswell, an organisation which aims to raise the voice of people with a lived experience of homelessness through research and activism.SH has lived experience of homelessness. Whilst both researchers are female, SDS and SH have considerable experience of interviewing people experiencing homelessness across the gender spectrum.<br>JB is a male Master's Psychology graduate and Research Fellow, with experience of working in a drug and alcohol recovery charity. BH and CS are female senior researchers in the specific area of homelessness and palliative care. They conceptualised the research and provided senior oversight throughout. JK, SK, PB and GD are members of the lived experience group for this project. MP is the Lived Experience Lead for Pathway, the homeless health charity which is supporting the lived experience group throughout the programme.<br><br>An interpretivist approach guided this study, recognising that individuals interpret and make meaning of their experiences in diverse ways. Our analysis aimed to understand how participants constructed meaning around questions related to the health and support for people with declining health in the context of homelessness, rather than to identify a single underlying reality.<br><br>This article has been written with both academic **and** operational audiences in mind as we feel that the findings will be particularly relevant for commissioners of homelessness services and health, housing and social care professionals.
Beyond issues of sample composition, the interviewer-participant relationship also warrants further consideration. The interviewers (a researcher and a co-researcher with lived	Whilst both researchers are female, SDS and SH have considerable experience of interviewing people experiencing homelessness across the gender spectrum.<br><br>The purpose of the interviews was initially to inform a larger community of practice-based intervention aiming to improve multiprofessional support for people experiencing homelessness who may have palliative care needs. The topic guide reflects these aims. This was highlighted to participants as part of the informed consent process. Comments about people's own health or

experience) appear to be female, while most participants were men.<br><br>This creates a complex social dynamic that may have influenced trust and rapport, disclosure of sensitive experiences, and the ways in which topics such as health, trauma, violence, and substance use were discussed during the interviews. It may also have shaped how participants chose to present their experiences and how particular issues were explored in the interview setting.	situations, were shared spontaneously and were not the result of direct questions about their own experiences, but rather what they felt health and social care professionals should know about supporting people in temporary accommodation.
Given the study's emphasis on lived experience involvement and co-production, a more explicit reflection on these relational dynamics would strengthen methodological transparency and provide greater insight into how knowledge was co-produced throughout the research process.	We have given more details on how we have interacted with and supported our lived experience steering group. We have also added more detail to the reflexivity section.
**Analytical approach and co-production**<br>While the study is presented as using reflexive thematic analysis, several themes appear primarily descriptive	We have reduced the number of quotations and increased the amount of analytic narrative.<br><br>We believe that the quotes remaining in the manuscript are powerful and compliment the themes described.

and topic-oriented rather than organised around broader interpretive concepts. The findings are richly illustrated with participant quotations, but the analytic narrative remains relatively limited.	
The manuscript would benefit from making the interpretive work of the researchers more visible, including how patterns of shared meaning were developed and how themes move beyond topical categorisation toward more conceptual insights.	Reflexive practices, including memo writing, discussion and iterative engagement with the data, supported transparency and analytic depth. An inductive approach to analysis was taken. The researcher positioning section of the paper has been expanded.
Furthermore, the manuscript highlights co-production and lived experience as central strengths. However, it remains unclear how these were operationalised in practice throughout the study. While reflexivity and author contributions are described, the relationship between these sections is not fully coherent.	We have added more information to the paper describing the Lived Experience Group that supports the wider project that this work is part of.<br><br>The IMPROVE project has a volunteer 5-person Lived Experience steering group which is supported by Pathway, an organisation which helps people experiencing homelessness to get involved in research and policy work. Within Pathway is a directly employed Lived Experience Programme Manager (MP) who supports the group to engage, providing practical and emotional support. The group members have met 1-2 monthly during the programme to contribute to the research process e.g. developing the questions, and discussing the emergent themes.<br><br>As part of the programme they also receive bi-monthly reflective practice provided by a psychologist with significant experience of working with people experiencing homelessness, and bi-monthly 1:1s are also provided by a member of the research team. Group members receive incentive vouchers for all their engagement.
It is unclear:<br><br>• How contributors with lived experience were involved in coding, theme and sub	The themes and analysis of these interviews were extensively discussed and iterated with members of the group, who are co-authors of this paper. For this set of interviews, the group members were not involved in the initial coding of the data, but they will have an opportunity to do this later in the programme.

theme development, and interpretation	
• How analytic decisions were made	Transcripts were analysed by two researchers – SDS and JB - using the Braun and Clarke reflexive thematic analysis approach(22). An inductive approach to analysis was taken. This involved initial familiarisation with the data through reading and re reading transcripts. Initial codes were generated in NVivo. These were then refined into candidate themes through the identification of shared patterns and meanings. These themes were then reviewed in relation to the coded data and the full dataset, in discussion with SH, BH and CS. Discussion with JK, SK, PB, GD and MP then helped to further refine the themes. These were then named and defined. Reflexivity was central throughout. Reflexive practices, including memo writing, discussion and iterative engagement with the data, supported transparency and analytic depth. SDS and JB wrote up the findings, but everyone had a chance to contribute and edit the manuscript. The process was highly collaborative.
• How disagreements or alternative interpretations were handled	The process was highly collaborative.
• How contributions were integrated into the writing process	SDS and JB wrote up the findings, but everyone had a chance to contribute.
In addition, greater detail regarding the lived experience steering group's involvement would be valuable. For example, information about the frequency and duration of meetings, the stages of the study at which the group was involved, and the nature of their contributions to analysis, interpretation, and manuscript development would help readers better understand the extent and influence of the	The group members have met bi monthly during the programme to contribute to the research process e.g. developing the questions, and discussing the emergent themes.<br><br>As part of the programme they also receive bi-monthly reflective practice provided by a psychologist with significant experience of working with people experiencing homelessness, and bi-monthly 1:1s are also provided by a member of the research team.

co-production process.	
The manuscript would also benefit from greater transparency regarding practical aspects of lived experience involvement, including whether members of the lived experience group received financial remuneration for their contributions, the basis on which this was provided, and whether any formal employment or consultancy arrangements were in place.	Both participants and members of the lived experience group received vouchers for all their engagement at a rate of £20 an hour. This has been added to the manuscript.
Greater transparency regarding who was involved at different stages of the analysis, how the analytic process evolved, and how decisions were reached would strengthen the credibility and rigour of the study.	Transcripts were analysed by two researchers – SDS and JB - using the Braun and Clarke reflexive thematic analysis approach (22). An inductive approach to analysis was taken. This involved initial familiarisation with the data through reading and re reading transcripts. Initial codes were generated in NVivo. These were then refined into candidate themes through the identification of shared patterns and meanings. These themes were then reviewed in relation to the coded data and the full dataset, in discussion with SH, BH and CS. Discussion with JK, SK, PB, GD and MP then helped to further refine the themes. These were then named and defined. Reflexivity was central throughout. Reflexive practices, including memo writing, discussion and iterative engagement with the data, supported transparency and analytic depth. SDS and JB wrote up the findings, but everyone had a chance to contribute. The process was highly collaborative.
**Results**<br>The results section is clearly structured and presents rich qualitative data. However, quotations at times appear to carry much of the interpretive burden. Additional analytic	We have reduced the number of quotes, and added more analytical commentary.

commentary would help clarify how the authors moved from individual accounts to broader patterns of shared meaning.	
**Discussion**<br><br>Need for a more integrated analytical narrative.<br><br>The discussion closely follows the structure of the results, addressing each theme in turn. While this approach is common, it leads to a fragmented reading experience and limits the development of a cohesive interpretive argument.<br><br>The discussion would benefit from identifying a smaller number of overarching interpretive insights that cut across themes, rather than discussing each theme largely in isolation. Linking methodological limitations to interpretation.	We have revised the discussion, taken out the headings and added an implications for practice section.<br><br>It is important to note that this isn't an article written primarily for an academic audience. It is written with the purpose of being able to be directly useful and usable by service providers and commissioners.<br><br>We have added further information about the limitations of the work.
The discussion would also benefit from more explicitly linking methodological issues (e.g. gender imbalance, interview dynamics, descriptive analysis) to the interpretation of findings. This would	We have added more information into the discussion on the limitations of the findings:<br>The number of people and services participating in the research is small, the number of women interviewed was small, and all interviewees were White British. As such the results may not be fully representative of a wider group of hostel and homelessness service users, particularly those with no eligibility for housing support in the UK. With regards to women, the smaller numbers do reflect the common gender balance seen in many homelessness

increase analytical transparency, clarify the scope and limits of the conclusions, and strengthen the credibility of the study.	services, but it is noted that women have been found to be more likely to be 'hidden homelessness'.<br>These findings should be interpreted in light of the fact that all three participating hostels were large scale settings. There has been a shift towards reducing the size of hostels in recent years to combat some of the above issues . It is also important to note that interviewees frequently recognised the commitment of hostel staff, who were perceived to be working hard to support residents despite high workloads and system challenges. While these contextual factors are important, they do not negate the concerns of interviewees.
**Literature and referencing**<br>Engagement with PPI and co-production literature<br>Given the central role of lived experience in the study design and interpretation, there is limited engagement with the Patient and Public Involvement (PPI) and co-production literature. Stronger theoretical positioning in relation to this body of work would contextualise the methodological approach, highlight the study's contribution, and strengthen its conceptual grounding.	We have put more in about the underpinning role of PPI and Co-production throughout the article.
Precision and alignment of citations There are instances where references appear broad or not fully aligned with the claims being made. Improving the precision of citations and ensuring clear alignment between claims and supporting	The reference section has been reviewed and updated.

evidence would strengthen the manuscript's rigour.	
Recommendations	
• Strengthen methodological transparency regarding reflexive thematic analysis, reflexivity, and co-production.	We have done this
• Reflect more explicitly on researcher positionality and the implications of the gender imbalance in the sample.	We have done this
• Develop a more interpretive and conceptually integrated analysis.	We have done this
• Reorganise the discussion to provide a clearer overarching analytical narrative	We have done this
. • Link methodological limitations more explicitly to the interpretation of findings.	We have done this
• Strengthen engagement with relevant PPI and co-production literature.	We have done this
• Improve the precision of referencing and alignment between claims and supporting evidence.	This has been amended.

VERSION 2 - REVIEW

**Reviewer**	**1**
**Name**	**Robinson, Jackie**
**Affiliation**	**University Of Auckland**
**Date**	**13-Aug-2026**
**COI**	

Exploring the views of people experiencing homelessness regarding their health and housing support, and their perspectives on deteriorating health and end of life support: a thematic analysis. BMJ Open August 2026

Thank you for the opportunity review this interesting article again. Whilst the manuscript has improved significantly there are a few things that still need to be addressed.

Title: I note this has been revised in response to a request from the Editor to include the study design. Thematic analysis is not the study design. The study design is more aligned with a qualitative exploratory design.

Abstract: please state clearly the objective of the study. As the abstract is currently written the objective section contains information about the study design and the findings.

Limitations – the authors have clearly stated the study limitations at the beginning of the article and discussed these more fully in the discussion however the fact that all participants were White British needs to be included and discussed more fully. Ethnicity particularly for migrants and indigenous peoples are overrepresented in many homeless communities around the world therefore this is an important limitation.

Introduction: More detail and reference to the international literature is needed to provide strong rationale for the study. At times statements are made which are not referenced e.g. "due to the high level of complexity and unmet need, mainstream palliative care services such as hospices, community nursing services and General Practitioners can be unsure how to support people who are experiencing homelessness." This is a significant statement to make and requires a reference.

Whilst I acknowledge the authors comments in response to my request to add more detail about the UK palliative care system, I still think this is an important point to include and could be relatively brief. It would provide rationale for the study in that the current palliative care model does not meet the needs of the homeless community and highlight the need for new and unique ways of providing palliative care for this population which I assume is what IMPROVE is trying to do?

More detail about the IMPROVE study has been added to the introduction and provides really good context for the current study, thank you. However, I think it requires a section of its own in the methodology section. In addition, included in the introduction are aspects of the study design which do not belong in the introduction. Am I correct in assuming that this study was undertaken to revise the IMPROVE intervention? This is still somewhat unclear.

Consider moving the study aims to the end of the introduction and before methodology section. Positionality section is really good and provides detail on the lenses in which the data was being analysed and interpreted.

Data analysis – please reference the approaches used such as inductive analysis, reflexivity and its contribution to transparency and rigor.

Findings – I don't quite understand the comment about fewer people who are non-White British are eligible for Local Authority funding support. Is this because they are no British Citizens?

One quote for each point made is usually a requirement – frequently there are two or more.

At times I wondered how the identified themes were specifically answering the research questions (apart from the last two themes which are quite specific about what participants wanted service providers to know). However, the discussion helped to more strongly link the themes back to the study questions.

**Reviewer**	**2**
**Name**	**Mattsson, Elisabet**
**Affiliation**	**Ersta Sköndal Bräcke University College**
**Date**	**10-Jul-2026**
**COI**	

Thank you for your thorough revisions. I appreciate the considerable effort the authors have made to address my comments. The manuscript has been substantially strengthened, particularly regarding methodological transparency, reflexivity, researcher positioning, participant recruitment and the analytic approach. I believe my major concerns have been satisfactorily addressed and have no further substantive comments.

VERSION 2 - AUTHOR RESPONSE

Reviewer 2
Title: I note this has been revised in response to a request from the Editor to include the study design. Thematic analysis is not the study design. The study design is more aligned with a qualitative exploratory design.

The title has now been changed to the following : Exploring the views of people experiencing homelessness regarding their health and housing support, and their perspectives on deteriorating health and end of life support: a qualitative analysis

Abstract: please state clearly the objective of the study. As the abstract is currently written the objective section contains information about the study design and the findings.
The abstract has been updated and a clear objective included.

Limitations – the authors have clearly stated the study limitations at the beginning of the article and discussed these more fully in the discussion however the fact that all participants were White British needs to be included and discussed more fully.
Ethnicity particularly for migrants and indigenous peoples are overrepresented in many homeless communities around the world therefore this is an important limitation. The fact that all interviewees were white British is already included in the strength and limitations section at the start of the manuscript and we have highlighted this also in the discussion section. We would acknowledge the fact that all participants being White British is slightly surprising, given that fact that in the UK homelessness populations are generally between 67-75%. However, we are just reporting the sample that presented themselves to us. The finding could simply be coincidental; however this is a learning point for us to ensure in future that the opportunity to be interviewed is advertised as inclusively as possible e.g. finding out in advance any potential language barriers that could exist to understanding posters or being offered the opportunity by key workers. The lead author of this article has experience of working in indigenous reservations in Australia. From her observations the cultural differences across ethnicities are maybe less marked in the UK, than in countries with indigenous populations. We appreciate that in some countries, indigenous populations are highly overrepresented in homeless communities. In this study we recruited people who were living in hostel or temporary accommodation. In order to be eligible to access these services, people are required to have recourse to public funds. People who do not have recourse to public funds are more likely to be from other ethnic backgrounds. We acknowledge that experiences of residents of hostels and temporary accommodation are likely to be different than people in other situations, but this was the focus of our paper. Whilst we were not purposively sampling based on ethnicity, in future this could be something that we could consider within the hostel setting.

Introduction: More detail and reference to the international literature is needed to provide strong rationale for the study.
Additional international references have been added to the introduction

At times statements are made which are not referenced e.g. “due to the high level of complexity and unmet need, mainstream palliative care services such as hospices, community nursing services and General Practitioners can be unsure how to support people who are experiencing homelessness.” This is a significant statement to make and requires a reference.
References have now been added to support this statement

Whilst I acknowledge the authors comments in response to my request to add more detail about the UK palliative care system, I still think this is an important point to include and could be relatively brief. It would provide rationale for the study in that the current palliative

care model does not meet the needs of the homeless community and highlight the need for new and unique ways of providing palliative care for this population which I assume is what IMPROVE is trying to do?
In response to this point, we have added a short sentence about how palliative care is delivered in the UK.

More detail about the IMPROVE study has been added to the introduction and provides really good context for the current study, thank you. However, I think it requires a section of its own in the methodology section. In addition, included in the introduction are aspects of the study design which do not belong in the introduction. Am I correct in assuming that this study was undertaken to revise the IMPROVE intervention? This is still somewhat unclear.
An additional section has been added to the methodology section to give more information about IMPROVE and how the data presented in the paper relates to it.

Consider moving the study aims to the end of the introduction and before methodology section. Positionality section is really good and provides detail on the lenses in which the data was being analysed and interpreted.
This section has now been moved to the end of the introduction

Data analysis – please reference the approaches used such as inductive analysis, reflexivity and its contribution to transparency and rigor.
Additional references have been added which outlines the inductive nature of our approach to the analysis
Braun, V., and V. Clarke. 2022b. Thematic analysis a practical guide. London: Sage
And also about reflexivity:
Olmos-Vega, F. M., Stalmeijer, R. E., Varpio, L., & Kahlke, R. (2023). A practical guide to reflexivity in qualitative research: AMEE Guide No. 149. Medical Teacher, 45(3), 241–251. https://doi.org/10.1080/0142159X.2022.2057287

Findings – I don’t quite understand the comment about fewer people who are non-White British are eligible for Local Authority funding support. Is this because they are no British Citizens? We have removed the sentence that you are referring to as we agree that what we were trying to express is a little complicated. Instead, we have added the following to the strengths and limitations section of the discussion: "All participants were self-selecting which could have introduced a bias in the sample. As such the results did not capture the experiences of people from a diverse range of ethnic backgrounds"

One quote for each point made is usually a requirement – frequently there are two or more.
We would prefer to keep all remaining quotes

At times I wondered how the identified themes were specifically answering the research questions (apart from the last two themes which are quite specific about what participants wanted service providers to know). However, the discussion helped to more strongly link the themes back to the study questions.

The themes that have been included reflect what was most important in participants

narratives.
We are glad that the discussion helped to link the themes back to the study questions.
